# Differences in Reproductive Success in Young and Old Females of a Long-Lived Species

**DOI:** 10.3390/ani11020467

**Published:** 2021-02-10

**Authors:** Amalia Segura, Roberto C. Rodriguez-Caro, Eva Graciá, Pelayo Acevedo

**Affiliations:** 1 Instituto de Investigación en Recursos Cinegéticos, IREC (CSIC-UCLM-JCCM), Ronda de Toledo, 12, 13071 Ciudad Real, Spain; pelayo.acevedo@uclm.es; 2 Applied Biology Department, Universidad Miguel Hernández, Avda. de la Universidad sn. Edificio Torreblanca, 03202 Elche, Spain; r.rodriguez@umh.es (R.C.R.-C.); egracia@umh.es (E.G.); 3 Department of Zoology, University of Oxford, Oxford 01865, UK

**Keywords:** interannual variation, maternal characteristics, offspring fitness, recruitment, survival, *Testudo graeca*

## Abstract

**Simple Summary:**

A central goal of life history studies is documenting traits related to reproduction. In long-lived species, older individuals might have traits that enhance reproductive success. However, survivorship at young life stages is usually unknown, which blurs the basic understanding of how female traits influence recruitment and the relevance of climate variation. Our study, which was based on the spur-thighed tortoise, demonstrated that (i) maternal age and climatic conditions influence the recruitment, (ii) older females have greater offspring numbers, greater survival and smaller size and mass than younger females and (iii) severe climatic conditions—low rainfall and temperature—reduce the number of surviving offspring despite their increased size. We discuss deeply how the maternal age and climatic conditions might affect population dynamics of long-lived species and recommended long-term studies of reproductive parameters and appropriate conservation actions.

**Abstract:**

Long-lived species are particularly interesting for investigation of trade-offs that shape reproductive allocation and the effective contribution to the next generations. Life history theory predicts that these species will buffer environmental stochasticity via changes in the reproductive investment, while maintaining high adult survival rates. The spur-thighed tortoise was selected as a case study in order to investigate the relationship between the linked maternal characteristics (size and age) and related traits in their hatchlings. We tracked naturally emerging hatchlings from young and old females under semi-natural conditions to test variations in hatchling numbers, body mass, size and survival over two years. We used linear mixed-effect models to analyze variations in hatchling body mass and size, and a mark–release–recapture framework to model their survival. Our study illustrates that old females of long-lived species have greater offspring numbers, greater survival and smaller size when compared with those of young females. The interannual variability evidenced the reduced offspring number and survival in the lower autumn rainfall and spring mean temperature year. Our results highlight the role of maternal age and climatic conditions in the population dynamics and the need for long-term studies of reproduction traits for designating adequate conservation strategies.

## 1. Introduction

Variation in stage-specific survivorship—e.g., young life stages—along with reproductive success, may shape the variability of life histories and, therefore, evolutionary changes [1,2]. Long-lived species are characterized by delayed maturity, high adult survival and low and variable recruitment rates [3,4,5,6]. They are generally classified as “slow metabolic rate species”, particularly prone to being affected by climate change, despite their ability to buffer certain environmental stochasticity [7]. Life history predicts that their investment in reproduction might be altered in response to changes in environmental conditions, while maintaining high adult survival rates [8]. This investment is dependent upon an orchestration of trade-offs that affect female fitness (linked to body condition) and comprises parental and offspring survival, as well as current and future reproduction [9,10]. For example, both theoretical and empirical studies have shown that offspring fitness is highly sensitive to changes in maternal size and age [9,10]. The benefits of recruiting as early as possible increases the number of lifetime reproductive attempts, which also increases fitness by shortening generation time [11], but it must be balanced with the associated costs as regards reduced survival and accelerated senescence in old age [12,13,14,15]. In addition, offspring lifespan decreases with increased parental age (known as the Lansing effect [16,17]), although very little is known about how parental lifespan influences offspring lifespan. Life history theory also assumes a trade-off between the number and size of offspring and the relationship between offspring size and survivorship, such that maternal fitness is optimized through an investment strategy maximizing the number of offspring that survive to sexual maturity [18]. However, in spite of the interest in this field, very few experimental studies have provided sound results for the offspring size and fitness relationship (hereafter “offspring size/fitness”) and survival in vertebrates [8,9,19,20]. The low detectability of breeders or newborns and juveniles [21,22] and the limited long-term studies play a part in this sense [15]. Studying recruitment patterns and how they are affected by environmental conditions is thus essential to determine demographic traits, to model and predict local distributions and to identify causal factors affecting long-term population dynamics [23]. This is especially relevant within the anticipated scenario of increased environmental stochasticity as a result of climate change [24,25]. 

Among long-lived species, chelonians are a particularly interesting species for investigation of the relationship between offspring size/fitness and the influence of maternal size and age. In tortoises and turtles, as in other ectothermic vertebrates, there is a strong intraspecific positive correlation between clutch size and maternal body size, e.g., [26]. In Testudines, in particular, clutch enlargement can be achieved at the expense of offspring size, and there is a negative correlation between clutch size and egg size [27,28]. Linked to this, younger females tend to produce fewer eggs than older ones that used to be bigger, e.g., [29]. Unfortunately, there is a knowledge gap in terms of both offspring (hereafter hatchling “first year”) survival and fitness and the factors that affect their susceptibility to environmental conditions, largely because of the secretive nature of the hatchlings and the difficulties involved in their tracking with the current available methods [30,31]. 

In this work we used the Mediterranean spur-thighed tortoise (*Testudo graeca*; Linnaeus, 1758) as a case study of a long-lived animal in order to investigate the relationship between the linked maternal characteristics size and age (hereafter maternal characteristics [29,32]) and related traits in their hatchlings (number, growth and survival). We selected a population located in Maamora forest, an anthropogenic cork oak forest located in Northern Morocco that is considered to be close to the optimum niche of the tortoise distribution [33]. This *T. graeca* population has been recognized as one of the densest documented to date [34]. We studied for two six-month periods the influence of maternal characteristics on the survival and phenology of hatchlings in their first year in a protected area, which is not susceptible to pet trade. Our specific objectives were (i) to describe the biometry of the hatchlings after emergence from short/young and longer/old females (hereafter “young” and “old”, respectively), (ii) to analyze changes in body size and mass of hatchlings in their first six months and (iii) to estimate the survival of hatchlings considering the maternal characteristics and interannual variability. 

## 2. Methodology

### 2.1. Study Site

The study was conducted in an area of low elevation (72–185 m a.s.l.) sandy soil in Maamora forest (Northwest Morocco; 34°02′54.19′′ N, 6°27′19.24′′ W). The climate is Mediterranean, with hot and dry summers, and the annual range of average rainfall is from 300 to 500 mm. Maamora forest is dominated by cork oak trees, *Quercus suber,* with scattered endemic wild pear, *Pyrus mamorensis,* wild olive *Olea europaea*, green olive *Phyllirea latifolia* and mastic *Pistacia lentiscus* and a sparse understory of bush and shrub species such as Mediterranean broom *Genista linifolia, Cytisus arboreus, Stauracanthus genistoides*, dwarf palm *Chamaerops humilis*, French lavender *Lavandula stoechas,* sage-leaved rockrose *Cistus salviifolius, Halimium halimifolium* and *Thymelaea lythroides.*

The study itself took place on private land where spur-thighed tortoise have not been exploited for the pet trade (protected for >10 years) and where undergrowth is well represented compared to other, unprotected sites in Maamora forest (for further details see [34]).

### 2.2. Mediterranean Spur-Thighed Hatchlings

**Figure 1:**
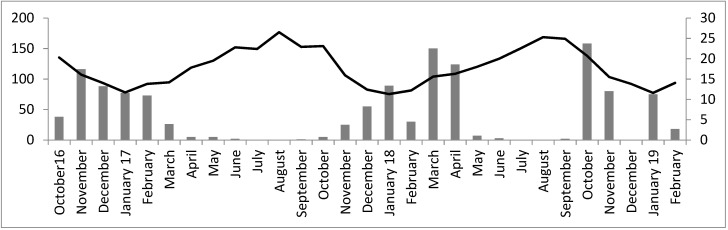
Climatic diagram: in black the precipitation (mm) and in grey mean average temperature (°C).

The study was carried out over two six-month periods (September–February, 2017/18 and 2018/19). Both average temperature (°C) and rainfall (mm) were recorded monthly from October 2016 to February 2019 (corresponding with the tortoise mating and incubating processes) from a meteorological station in the study area (Kenitra; see Figure 1).

In January 2017, an area of 2500 m^2^ was divided into four smaller areas of 625 m^2^ each that were characterized by a diversity of autochthonous vegetation, including sage-leaved rockrose (actively selected by females for nesting). The environmental conditions in the selected areas were similar (e.g., vegetation to hide from predators and food resources for growing; see electronic supplementary material (ESM) S1, Table S1). Those areas (hereafter “female-age areas”, *n* = 4) were fenced off in order to subsequently house females for egg-laying and hatchlings for survival and growth monitoring. All the areas had been checked to ensure the absence of adult tortoises within them. In addition to the female-age areas, an adjacent non-fenced area (hereafter “adjacent area”) located <10 m apart from the female-age areas with similar characteristics of size and vegetation was monitored. In April of 2017 and 2018, 10 young (130–160 mm carapace length, CL, and 14–20 years) and 10 old females (180–200 mm CL and 23–31 years; see EMS Table S2 for further details about female characteristics) per year were collected from the surrounding areas (12 ha) and released into different areas (e.g., separating young from old females and using only two areas per year), where they were left to nest naturally. Female classes of young females were based on sexual maturity (ranging from 100.5 to 114.6 mm CL [35,36]) and old ones on both size and age studies of *T. graeca.* The size is highly correlated with age, and the age estimation was carried out according to ring counts and corrected by assuming an underestimation of one year every four years [32]. The maximum lifespan in wild populations of *T. graeca* is around 30 to 35 years [29,37]). In June of each study period, after laying several clutches inside the female-age areas through the reproduction season [38], each female was returned to the location in the surroundings from which they had initially been taken. Emerging hatchlings were marked with non-toxic paint (using individual codes) and tracked, wherever possible, from their emergence from the nest in September until they were found dead or until the end of the study, the following spring. The capture–recapture framework, which allows for dealing with imperfect detection, was used to estimate tortoise survival rates and record weight and size, e.g., [39,40,41,42]. The frequency of tracking for the survival monitoring was done during several visits in 15-day periods each month. At the beginning of the study (September and October), we increased the tracking effort to mark all the individuals (see EMS Figure S1). All hatchlings were both weighed (g; body mass) and carapace length measured (mm; CL) monthly after recapture, mostly the last week of the month. Mortality was recorded on the day of occurrence, or the day after, and the cause of death was inferred and recorded whenever possible. Differences between the initial number of hatchlings per tortoise from young and old females were tested by the chi-squared test of independence.

### 2.3. Hatchling Body Mass and Size Analysis

The changes in body size and mass were tested using linear mixed-effect models parameterized in R software [42]. Body size and mass of each measured individual were the response variables, individual was used as random factor and month (September to February), period (2017/18 and 2018/19) and female age (young/old females) as fixed factors.

Measurements of body mass and size were restricted in 2017/18 to 14 and 37 hatchlings from young and old females, respectively, and 3 and 18 in 2018/19, respectively (ESM Table S3 for further details).

Stepwise backward procedures were used to model selection and were based on Akaike’s information criterion adjusted for small sample sizes (AICc [43]). The selection stopped when removing an additional predictor did not improve the model in terms of AIC and did not reduce AIC in more than 2 units. Moreover, the relative support for each model was estimated using Akaike weights (*ω*).

### 2.4. Modelling Hatchling Survival

We used a mark–release–recapture framework to model the survival and resighting probability—the probability of observing a marked tortoise—using the software program MARK [44]. Resightings were grouped into two different periods (2017/18 and 2018/19; hereafter “periods”). The data were collected fortnightly, the second and fourth week of the month in 7-day time intervals (from the last week of September to the second week of February; hereafter “time intervals”). As such, survival rates have a temporal resolution of two weeks. 

The notation used for survival models follows Lebreton et al. [39]. For our initial model we used (φ_[female age, period]_ ρ_[female age, period, time interval]_), including both survival (φ) and resighting probability (ρ). Two different survival models were developed according to the available data: (1) to determine differences due to maternal characteristics (hatchlings from young and old female areas), which included female age and time interval as factors, and (2) to determine differences due to interannual variability, which included period and time interval as factors. Due to the quantity of data, the first analysis was restricted to 45 hatchlings (14 from young females and 31 from old females) of the 51 hatchlings marked in the period 2017/18, which corresponded to the selected time intervals, and the second was carried out just for hatchlings from 44 hatchlings (30 in 2017/18 and 14 in 2018/19 period) of the 55 initially marked, which corresponded to the selected time intervals of old females in the two periods. Particularly, in the second model, hatchlings from young females were not included in the analysis due to their low number in the 2018/19 period (*n* = 3).

All models were parameterized using the logit-link function. Survival was considered to be constant for all individuals or to change as a function of female age/period, and resighting probabilities were also considered to be either constant for all individuals or to change as a function of female age/period and/or time interval (with interaction and additive effect). Model selection was based on AICc and Akaike weights [43]. Additionally, for each model we calculated the Akaike weights, *ω*, as an index of its relative plausibility [44]. We used *ω* to estimate survival rates using model average. Goodness-of-fit tests were used to assess the fit of the global models to the resighting histories, under the assumptions of the Cormark–Jolly–Seber model [45], and were calculated by building specific contingency tables for each recapture occasion using program U-CARE [46]. No significant deviation of these assumptions was found for the differences between female age areas in 2017/2018 (χ^2^ = 10.91, *p* = 0.99) or between periods when limited to hatchlings from old females (χ^2^ = 10.10, *p* = 0.99).

## 3. Results

### 3.1. Description of Hatchling Biometry from Emergence and Environmental Differences

The first signs of hatchlings were observed in the second week of September in 2017 and in the fourth week of September in 2018, and were irrespective of maternal characteristics. Seventy-two hatchlings were tracked in the female-age areas during the two-year study period: 51 in 2017/18 and 21 in 2018/19. There were significant differences between the number of newborns from old and young females in both periods (χ^2^ = 7.07 *p* < 0.05 *n* = 51 in 2017 and χ^2^ = 7.57 *p* < 0.05 *n* = 21 in 2018; see Table 1). It was consistently higher than for younger females across two years (2.7 ± 1.3 vs. 0.9 ± 0.7 hatchlings per old and young female, respectively). Significant differences between years were observed, with 50% fewer newborns in 2018 than in 2017 (χ^2^ = 4.97 *p* < 0.05 *n* = 17 for young females and χ^2^ = 4.43 *p* < 0.05 *n* = 55 for old females; see Table 1). None of the hatchlings from the young female areas were found dead, although in 2017/2018 two from the old female area died, one a couple of days after being born and the other in January presumably following injuries it had received to the nose. However, 10 hatchlings were found dead (six in 2017 and four in 2018) in the adjacent area: five hatchlings had been predated by common ravens *Corvus corax*, three had been trampled, presumably by livestock, and two deaths occurred in winter after heavy rains.

The autumn rainfall in 2016 (October–December previous to the first incubation) was higher when comparing with autumn 2017. Nevertheless, spring rainfall in 2017 (April–June during the incubation) was lower when comparing with spring 2018 (see Figure 1). The temperature in spring 2017 was higher than spring 2018 (see Figure 1). 

### 3.2. Hatchling Body Mass and Size 

**Figure 2:**
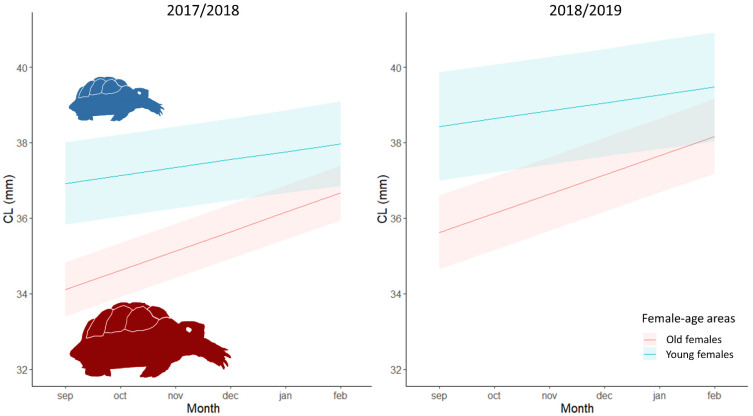
Relationship between body size (carapace length, CL, in mm) and months for hatchlings from young females (in blue) and old females (in red) during the study period. Body size was calculated with the linear mixed model that best fit the observed data, which include the interaction of month and female age, the additive effect of period and the random effect of the individual (Table 2). Lines represent the estimated mean values, and the shadow areas correspond to the confidence interval (95%).

**Figure 3:**
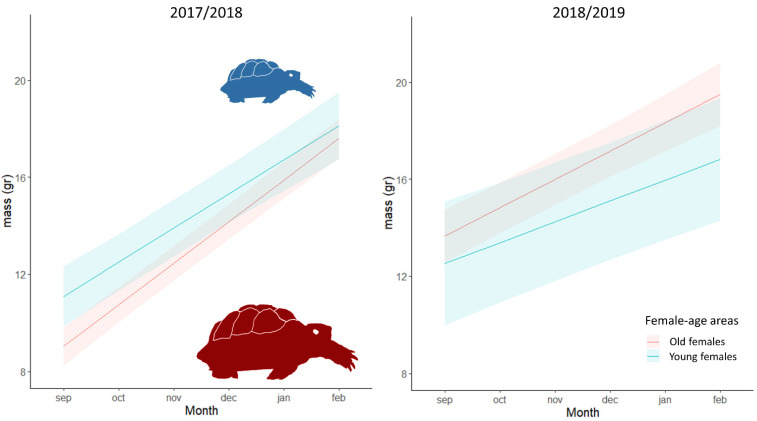
Relationship between hatchling body mass (in g) and months, of young females (in blue) and old females (in red) during the study period. Body mass was calculated with the linear mixed model that best fit the observed data, which include the interaction between month, female age and period and the random effect of the individual (Table 2). Lines represent the estimated mean values, and the shadow areas correspond to the confidence interval (95%).

Significant differences in both body size and body mass were found between female-age areas and periods. The most parsimonious model showed that hatchling body size was explained by the interaction of month and female age, with an additive effect of period (Table 2, ESM Table S4). Hatchlings from old females grew more than those from young females throughout the study period and were bigger in 2018/19 than 2017/18 (Figure 2). Similarly, the most parsimonious model showed that body mass was explained by the interaction between month, female-age area and period (Table 2, ESM Table S5). Nevertheless, the hatchling mass from old females in 2018/2019 was larger than that from young females when comparing with the previous period (Figure 3). 

However, despite body mass and size at and just after emergence being higher in hatchlings from young females, individuals of the two female-age areas had reached similar body sizes and mass at the end of the study period in February 2019 (Figure 2 and Figure 3).

### 3.3. Hatchling Survival

The best-fit models of the analysis of survival with respect to determining the impact of differences in maternal characteristics incorporated the female age in the survival estimates, and the resighting probabilities were estimated using the time interval and the additive effect of female age (Table 3, see ESM Table S6). According to Akaike weights, hatchling survival from model average (±; SE) was 0.85 ± 0.05 for those born from young females and 0.96 ± 0.02 for hatchlings born from old females. Average hatchling resighting probabilities were 0.38, ranging from 0.00 ± 0.00 to 0.77 ± 0.11, for young female hatchlings, and 0.30, ranging from 0.00 ± 0.00 to 0.68 ± 0.11, for those from old females. 

The best-fit model of the survival analysis to determine interannual differences incorporated female age in the survival and the interaction of the time interval with the period in the resighting probabilities (see Table 4, ESM Table S6). This model comprised 100% of Akaike weights, and hatchling survival was 0.94 ± 0.03 and 0.81 ± 0.06 in 2017/2018 and 2018/2019, respectively. Average hatchling resighting probabilities were 0.32 in 2017/18 and 0.36 in 2018/19. 

## 4. Discussion

Understanding the influence of maternal age and size, offspring size-fitness and climatic conditions on recruitment success is important for managing more effectively those populations through conservation strategies. Our study illustrates the important role that old females of long-lived species might play in population dynamics, i.e., the higher number and survival of their offspring from emergence to overwintering. The interannual differences, which might be linked to environmental conditions, increase even further the variability in recruitment success, with number and survival of offspring falling in more severe meteorological conditions (lower autumn rainfall and spring temperature). 

### 4.1. Maternal Characteristics and Offspring Fitness

In long-lived species, the relative reproductive rate hypothesis states that old individuals have traits that enhance reproductive success directly (e.g., clutch and offspring size) or indirectly through increased reproductive life compared to younger individuals. Mediterranean spur-thighed tortoises show delayed reproduction and low breeding success, which increases with age. According to our results, maternal characteristics influence recruitment in those older females that had greater numbers of newborns compared with young females [15,29]. Moreover, with regard to hatchling fitness, initial body mass and size did not limit survivorship at the end of hatchlings’ first spring, contradicting the bigger is better hypothesis, which states that initial higher mass and size give an advantage to hatchling survival of the first winter [47,48,49,50]. Indeed, the size of newborns from older females was lower than those from young females, although after overwintering, these hatchlings had reached a similar size as the originally bigger hatchlings from young females. Nevertheless, the higher mass of hatchlings from old females when comparing with those from young females (*n* = 3) in the second period pinpoints the need for further studies that will strengthen this hypothesis. In addition, it is possible that other mechanisms that favor small size in newborns at our site could be involved, such as some favorable survival-related performance attributes in young tortoises (e.g., smaller ones being faster [50] and more prone to self-righting as antipredator behavior [51]) or that size might be inversely proportional to another morphometric trait related to survival [47]. In addition, bearing in mind that the study comprises only the first year of their life, there is a possibility that, in accordance with the Lansing effect, in future years the lifespan of hatchlings from old females might be shorter than that of young ones. Finally, the mechanisms related to offspring size/fitness drivers and the effect of parental age on offspring lifespan are unknown and merit long-term studies to disentangle the life-history traits of long-lived species because they have a great impact on both evolutionary and ecological processes, from the individual to the population level [11,15,52,53]. On the other hand, those mentioned mechanisms should be included in chelonian conservation strategies, through headstarting programs, habitat management or even campaigns against pet collection and trade, which should focus on the important role of the lifetime reproductive output of older females.

### 4.2. Influence of Interannual Variation on Recruitment Success 

Variations among years (e.g., in environmental conditions and food availability) may strongly affect the recruitment process in long-lived species [54,55]. Acknowledging that our results are limited to hatchlings from old females, which might hinder information on young mature females, differences in the relationship number/size of the hatchlings between years point to the impact of climatic variables, particularly rainfall and temperature. Rainfall might alter maternal investment [56,57,58], clutch frequency and the percentage of adult females that reproduce in a given year [59], and temperature influences physiology, the rate of incubation time and posthatching survival [60]. Indeed, low rainfall in the months prior to nesting might reduce the number of gravid females as well as their fitness, thus conditioning and restricting the emergence and number of newborns. Hofmeyr et al. [61] documented an increase in newborn size associated with the unpredictability of rainfall. In the same vein, in the period with lower rainfall (autumn 2018), old females produced fewer hatchlings with higher size and mass (50%) than in the previous year, when autumn rainfall was higher. Indeed, autumn rainfall has been described to affect distribution and abundance in *T. graeca* [62]. Nevertheless, this higher investment in hatchling size and mass in order to reduce the physiological costs associated with worse environmental conditions (e.g., lower food resources) did not result in an increase of the survival during the period of lower autumn rain, e.g., [63]. In addition, warmer temperature in the incubating months favors shorter clutch phenophases and higher hatchling survival [64]. Thus, the less warm temperature in spring 2018 might have played a part in the lower survival of the hatchlings. 

Bearing in mind that climatic conditions are likely to vary between years, and the fact that our study only comprised two years, this maternal investment trade-off might reduce associated costs, resulting in lower recruitment success [56]. These climatic shifts are particularly important in arid and semiarid ecosystems such as the Mediterranean basin [65,66,67], where longer periods of droughts and heavy rains are expected [60], by increasing the threats to long-lived species, e.g., poorer body condition, lower survival and recruitment rates [56]. Therefore, forecasting the future responses of natural systems to changes in climatic conditions—particularly rainfall regimes—will help to determine the degree of resilience of the species and to implement mitigation strategies [24,68,69].

### 4.3. Increasing Descriptive Knowledge of Tortoise Hatchling Biometry 

Monitoring studies considering the phenology and survival of neonates following emergence of chelonian hatchlings are limited to date (five studied species [47,70,71,72,73]). The low detectability of tortoises in the hatchling stage (e.g., 0.30–0.38 detection probability for *T. graeca*), even in intensive field studies, is clearly a handicap. Nevertheless, hatchling survival at natural abundance levels may reflect natural ecosystem patterns [47] and is important information to have in order to estimate long-term population trends and causal factors that might limit tortoise populations [74].

Hatchling size, mass and survival, and therefore fitness, are related to foraging and to the quality of food resources [75]. In our study, the hatchlings seemed to have larger sizes and higher mass at the end of the spring season compared to their Spanish counterparts, which might be explained by the high volume and diversity of palatable herbs that characterize this Moroccan area. The survival of juvenile tortoises, particularly hatchlings, is influenced by their habitat because of variation in food abundance, susceptibility to predators and the environmental conditions specific to different habitats. Overall survival from emergence to overwintering in this study was higher in hatchlings from old females (56% when extrapolating fortnight survival) than their counterparts in Southern Spain (39% [31]). In our study, raven predation is a plausible threat for the hatchling population, as occurred in other populations of Maamora forest [22] and in other Testudinidae populations, where it is known to cause high mortality [72]. However, the effect of traffic and trampling by large ungulates found in our study, and as occurred in northern populations of *T. graeca* [31], is a minor cause of mortality. Finally, it is possible that the differences observed in hatchling fitness might also be a consequence of the optimal environmental conditions associated with this niche of the tortoise distribution [34], where higher recruitment rates might be more expected in core areas than in population border ranges. Further studies are required to disentangle the differences in tortoise recruitment across the niche of tortoise distribution.

## 5. Conclusions

Survivorship at young life stages is usually unknown, which blurs the basic understanding of how female traits influence recruitment and the relevance of climate variation. Our study provides evidences that maternal age and climatic conditions influence the recruitment in long-lived species. Our findings are expected to be useful in designing conservation strategies, which integrate both the lifetime reproductive output of older females and the climatic shifts. On the other hand long-term studies of reproductive parameters are needed to better understand the ecological effects of trait variations within long-lived species.

## Figures and Tables

**Table 1:** Successful clutch number per season (2017 and 2018) from young and old females and number of hatchlings per tortoise. Hatchling mean body size (mm) and body mass (g) with ranges (in brackets) after emerging.

Items		No Hatchlings/Tortoise	Hatchling Body Size	Hatchling Body Mass
2017	Young (*n* = 14)	1.4	38 (36–39)	11.5 (11–13)
	Old (*n* = 37)	3.7	34 (29–38)	9.8 (8–13)
2018	Young (*n* = 3)	0.3	37 (36–37)	12.7 (12–13)
	Old (*n* = 18)	1.8	36 (33–39)	14.6 (12–17)

**Table 2:** Summary and statistical parameters of the final models for size (carapace length) and body mass. The individual was considered as a random factor, month as a continuous variable (from September to February) and female age (young or old females) and period (2017/2018 and 2018/2019) as factors. See electronic supplementary material (ESM) Tables S4 and S5 for model selection. * represents the interaction between the variables.

Model	Variable	Estimate	Std. Error
Size	Intercept	29.53	0.48
	Month	0.51	0.03
	Female age (young)	5.51	0.89
	Period (2018/2019)	1.51	0.58
	Month * female age (young)	−0.30	0.06
Body mass	Intercept	−6.42	0.90
	Month	1.72	0.07
	Female age (young)	4.86	1.65
	Period (2018/2019)	9.56	1.65
	Month * female age (young)	−0.31	0.13
	Month * period (2018/2019)	−0.55	0.14
	Period (2018/2019) * female age (young)	−3.19	1.50

**Table 3:** Summary of hatchling survival models, including resighting probability (*ρ*) and survival probability (*φ*) and considering maternal characteristics for the 2017/2018 period. Female age and time interval were considered as fixed effect factors. Model selection was based on Akaike’s information criterion corrected for small sampling size (AICc); ΔAICc is the difference between the current model and the one with the lowest AICc value. Model weights (*ω*), number of parameters (nPars) and relative deviance (Dev.) are also shown. See ESM Table S6 for statistical parameters.

Model Specification	AICc	ΔAICc	*ω*	nPars	Dev.
*φ*_(e)_ *ρ*_(e + t)_	322.50	0	0.48	11	145.29
*φ*_(e)_ *ρ*_(t)_	323.20	0.70	0.33	10	148.48
*φ*_(.)_ *ρ*_(t)_	326.25	3.76	0.07	9	153.97
*φ*_(e)_ *ρ*_(e * t)_	326.39	3.89	0.07	16	135.91
*φ*_(.)_ *ρ*_(e + t)_	327.34	4.84	0.04	10	152.62
*φ*_(.)_ *ρ*_(e * t)_	331.10	8.60	0.00	15	143.39
*φ*_(e)_ *ρ*_(.)_	359.50	37.00	0.00	3	200.84
*φ*_(.)_ *ρ*_(.)_	363.50	41.00	0.00	2	206.95

Model notation: “t” = time interval, “e” = female age (hatchlings from young and old females), “+” = parallel variation (i.e., additive model), “*” = interaction.

**Table 4:** Summary of hatchling survival models, including resighting probability (*ρ*) and survival probability (*φ*) and taking into account interannual variability between the 2017/2018 and the 2018/19 periods. Model selection was based on Akaike’s information criterion corrected for small sampling size (AICc); ΔAICc is the difference between the current model and the one with the lowest AICc value. Model weights (*ω*), number of parameters (nPars) and relative deviance (Dev.) are also shown. See ESM Table S6 for statistical parameters.

Model Specification	AICc	ΔAICc	*ω*	nPars	Dev.
*φ*_(pe)_ *ρ*_(pe * t)_	236.80	0.00	1.00	14	112.30
*φ*_(pe)_ *ρ*_(.)_	252.57	15.77	0.00	3	155.86
*φ*_(pe)_ *ρ*_(t)_	252.77	15.96	0.00	11	136.69
*φ*_(.)_ *ρ*_(pe)_	253.06	16.26	0.00	3	156.35
*φ*_(pe)_ *ρ*_(pe)_	253.73	16.92	0.00	4	154.81
*φ*_(.)_ *ρ*_(t)_	253.89	17.08	0.00	10	140.46
*φ*_(.)_ *ρ*_(.)_	255.39	18.59	0.00	2	160.83
*φ*_(pe)_ *ρ*_(pe + t)_	255.48	18.68	0.00	12	136.67

Model notation: “t” = time interval, “pe” = period (hatchlings from 2017/2018 and 2018/2019), “+” = parallel variation (i.e., additive model), “*” = interaction.

## Data Availability

The datasets generated during and/or analyzed during the current study are available from the corresponding author on reasonable request.

## Supplementary Materials

The following are available online at https://www.mdpi.com/2076-2615/11/2/467/s1, S1: Description of the female-age areas, Table S1: Vegetation of the female-age areas, Table S2. Female body size (mm, carapace length; CL), body mass (g) and age (number of rings, Rodriguez-Caro et al. 2015). Table S3. Monthly average values (+ SD) of body size (carapace length CL; mm) and body mass (BM; g) of *Testudo graeca* hatchlings in Maamora forest. Table S4. Models of hatchling size (carapace length; CL in mm), using linear mixed-effects that included the individual as random factor. The fixed factors comprised month, considered as a continuum variable, period and female age. Model selection was based on Akaike’s Information Criterion corrected for small sampling size (AICc); ΔAICc is the difference between the current model and the one with the lowest AICc value; model weights (*ω*) and degrees of freedom (*df*) are shown. Table S5. Models of hatchling mass (in g), using linear mixed-effects that included the individual as random factor. The fixed factors comprised month, considered as a continuum variable, period and female age. Model selection was based on Akaike’s Information Criterion corrected for small sampling size (AICc); ΔAICc is the difference between the current model and the one with the lowest AICc value; model weights (*ω*) and degrees of freedom (*df*) are shown. Table S6. Summary and statistical parameters of the best models for maternal size/age (young and old) and the final model of interannual differences (2017/18 and 2018/19) including resighting probability (*ρ*) and survival probability (*φ*). Figure S1. Diagram of the monitoring effort exerted tracking hatchlings to estimate the survival rates (in blue sampling days). The study period encompasses six months, the end of September to February, during the two periods: a) 2017/2018 and b) 2018/2019. The effort was higher at the beginning of the study to mark all the new individuals when they were born and highly active and detectable. The data for the model analysis was collected fortnightly, and tracking individuals that were coincident in the same weeks were grouped to increase the sample size.
