# Supplementary material for: Differences in Reproductive Success in Young and Old Females of a Long-Lived Species
Source: Animals (Basel). 2021 Feb 10;11(2):467. doi: 10.3390/ani11020467 (PMC7916336; doi:10.3390/ani11020467)

animals MDPI

Supplementary Materials: Differences in Reproductive Success in Young and Old Females of a Long-Lived Species

Amalia Segura [1,*], Roberto C. Rodriguez-Caro [2,3], Eva Graciá [2] and Pelayo Acevedo [1]

[1] Instituto de Investigación en Recursos Cinegéticos, IREC (CSIC-UCLM-JCCM), Ronda de Toledo, 12, 13071 Ciudad Real, Spain; pelayo.acevedo@uclm.es
[2] Universidad Miguel Hernández, Avda. de la Universidad sn. Edificio Torreblanca, 03202 Elche, Spain; r.rodriguez@umh.es (R.C.R.-C.); egracia@umh.es (E.G.)
[3] Department of Zoology, University of Oxford, 01865 Oxford, UK
* Correspondence: amaliasegura@gmail.com; Tel.: +34-926295450

Citation: Segura, A.; Rodriguez-Caro, R.C.; Graciá, E.; Acevedo, P. Differences in Reproductive Success in Young and Old Females of a Long-Lived Species. *Animals* **2021**, *11*, 467. https://doi.org/10.3390/ani11020467

Academic Editor: Jean-Marie Exbrayat
Received: 30 December 2020
Accepted: 5 February 2021

Published: 10 February 2021

Publisher's Note: MDPI stays neutral with regard to jurisdictional claims in published maps and institutional affiliations.

Copyright: © 2021 by the authors. Licensee MDPI, Basel, Switzerland. This article is an open access article distributed under the terms and conditions of the Creative Commons Attribution (CC BY) license (http://creativecommons.org/licenses/by/4.0/).

S1. Description of the female-age areas for housing female egg-laying and hatchlings The location of the fenced areas was selected according with its representativeness in terms of vegetation composition. The vegetation in the four fenced continuous areas was similar in terms of cover (percentage) and richness in bush, scrub and herb (see table S1). Particularly the same species of bush, scrub and herbs (dominating the gramineae, leguminoseae and asteraceae families) were found in the four fenced areas. The design and construction of the fence was done according to tortoise characteristics. The fence was 1m high buried in the ground. The material of the fence was wood. To avoid trampling nests only a person checked the tortoise nests in August and September. In addition to avoid human inference in the experiment, only a person per day monitor the hatchlings in all the fenced areas.

Table S1. Vegetation of the four female-age areas.

	**Bush**		Scrub		Herb	
	**Cover**	***Richness**	Cover	*Richness	Cover	Richness
Old females 2017	20	2	50	3	30	27
Young females 2017	25	2	50	3	25	27
Old females 2018	20	2	55	3	25	25
Young females 2018	25	2	50	3	25	27

*Bush richness: *Genista linifolia* and *Olea europaea*; Scrub richness: C*hamaerops humilis, Cistus salviifolius* and Lavandula stoechas.

Table S2. Female body size (mm, carapace length; CL), body mass (g) and age (number of rings, Rodriguez-Caro et al. 2015).

Year	Young female				Old female			
	**Rings**	**Age**	**Size**	**Mass**	**Rings**	Age	Size	Mass
2017	15	19	152	778	22	28	190	1371
	16	20	160	816	19	24	185	1227
	14	18	143	564	18	23	180	1104
	12	15	130	464	20	25	188	1120
	11	14	135	529	22	28	197	1154
	15	19	161	863	23	29	194	1343
	15	19	145	677	19	24	181	1074
	13	16	139	537	22	28	185	1043
	15	19	153	803	18	23	183	1224
	13	16	140	560	22	28	192	1390
2018	11	14	133	481	19	24	180	1061
	11	14	135	451	22	28	191	1350
	12	15	136	516	21	26	200	1380
	15	19	150	466	20	25	185	1107
	11	14	135	504	18	23	188	1220
	14	18	145	690	25	31	195	1453
	15	19	161	746	23	29	198	1483
	13	16	140	598	19	24	183	1097
	13	16	155	868	18	23	181	1050
	16	20	163	913	22	28	190	1261

*Age has been calculated by correcting the number of rings, adding each four years one.

Table S3. Monthly average values (+ SD) of body size (carapace length CL; mm) and body mass (BM; g) of *Testudo graeca* hatchlings in Maamora forest.

	Young females					Old females				
		Body mass		CL			Body mass		CL	
	n	Mean	Range	Mean	Range	n	Mean	Range	Mean	Range
2017/18										
September	8	11.62±1.41	10-14	37.37±1.19	36-38	13	9.46±2.18	7-13	33.61±3.01	29-39
October	10	12±1.25	10-14	37.7±0.95	36-39	31	10.16±2.15	7-13	34.43±2.49	29-38
November	1	12.00		38.00		26	13.38±2.50	9-18	35.46±2.95	29-40
December	5	15.8±1.92	13-18	37.4±0.55	37-38	23	15±2.54	9-19	36.08±2.96	29-41
January	6	17.83±1.3	16-19	38±0.89	37-39	24	16.04±2.35	10-20	36.65±1.92	32-39
February	3	16.67±1.53	15-18	38.33±1.53	37-40	7	18.85±3.39	15-23	38.5±1.97	37-42
2018/19										
September						6	13.5±1.73	12-15	35.33±1.86	33-37
October	3	12.67±0.58	12-13	36.67±0.58	36-37	15	14.88±1.93	13-17	36.6±2-03	33-39
November	2	14.5±0.71	14-15	36.5±0.71	36-37	10	16.46±2.85	14-20	37.4±1.9	35-40
December	2	16±1.41	15-17	37.00		9	17±3.34	13-21	37.22±1.86	35-40
January	3	16.33±1.53	15-18	37.67±0.58	37-38	6	17.17±3.76	15-22	37.67±2.42	35-40
February	1	15.00		37.00		3	17.5±0.71	17-18	37.67±2.08	36-40

Table S4. Models of hatchling size (carapace length; CL in mm), using linear mixed-effects that included the individual as random factor. The fixed factors comprised month, considered as a continuum variable, period and female age. Model selection was based on Akaike's Information Criterion corrected for small sampling size (AICc); ΔAICc is the difference between the current model and the one with the lowest AICc value; model weights (ω) and degrees of freedom (*df*) are shown.

**Model specification[1]**	***df***	**AICc**	**ΔAICc**	ω
Month * Female age + Period	7	592.03	0.00	0.87
Month * Female age	6	597.12	5.09	0.07
Month * Female age * Period	10	597.29	5.26	0.06
Month + Female age * Period	7	606.51	14.48	0.00
Month + Female age + Period	6	610.61	18.58	0.00
Month + Female age	5	615.08	23.06	0.00
Month + Period	5	620.83	28.80	0.00

Month	4	622.92	30.90	0.00
Female age + Period	5	757.78	165.75	0.00
Female age	4	761.03	169.00	0.00
Period	4	766.97	174.95	0.00
Null	3	768.37	176.34	0.00

[1] models represented with "+" include variables in an additive way, while those represented with "*"include the interaction.

Table S5. Models of hatchling mass (in g), using linear mixed-effects that included the individual as random factor. The fixed factors comprised month, considered as a continuum variable, period and female age. Model selection was based on Akaike's Information Criterion corrected for small sampling size (AICc); ΔAICc is the difference between the current model and the one with the lowest AICc value; model weights (ω) and degrees of freedom (*df*) are shown.

Model specification[1]	*df*	AICc	ΔAICc	*ω*
Month * Female age * Period	10	848.78	0.00	0.99
Month + Female age * Period	7	857.81	9.03	0.01
Month * Female age + Period	7	863.10	14.33	0.00
Month + Period	5	864.00	15.22	0.00
Month + Female age + Period	6	864.12	15.34	0.00
Month	4	883.32	34.54	0.00
Month * Female age	6	884.08	35.30	0.00
Month + Female age	5	884.11	35.33	0.00
Period	4	1111.56	262.78	0.00
Female age + Period	5	1112.11	263.34	0.00
Null	3	1123.00	274.22	0.00
Female age	4	1123.70	274.92	0.00

[1] models represented with "+" include additive variables in an additive way, models represented with "*"include the interaction. .

Table S6. Summary and statistical parameters of the best models for maternal size/age (young and old) and the final model of interannual differences (2017/18 and 2018/19) including resighting probability (ρ) and survival probability (φ).

Model	Variable	Estimate	Std. Error	Lower	Upper
Maternal size/age ω = 0.48	$\varphi_{(e:\ young)}$	0.837	0.049	0.717	0.913
	$\varphi_{(e:\ old)}$	0.960	0.019	0.900	0.985
	$\rho_{(e:young,\ t1)}$	0.141	0.131	0.019	0.578
	$\rho_{(e:\ young,\ t2)}$	0.272	0.113	0.109	0.533
	$\rho_{(e:young,\ t3)}$	0.246	0.106	0.096	0.501
	$\rho_{(e:young,\ t4)}$	0.000	0.000	0.000	0.000
	$\rho_{(e:young,\ t5)}$	0.494	0.139	0.248	0.743
	$\rho_{(e:young,\ t6)}$	0.668	0.122	0.407	0.855
	$\rho_{(e:young,\ t7)}$	0.479	0.137	0.239	0.729
	$\rho_{(e:young,\ t8)}$	0.700	0.117	0.438	0.875
	$\rho_{(e:young,\ t9)}$	0.820	0.094	0.566	0.941
	$\rho_{(e:old,\ t1)}$	0.068	0.070	0.008	0.386
	$\rho_{(e:old,\ t2)}$	0.144	0.067	0.055	0.328
	$\rho_{(e:old,\ t3)}$	0.127	0.059	0.049	0.292
	$\rho_{(e:old,\ t4)}$	0.000	0.000	0.000	0.000
	$\rho_{(e:old,\ t5)}$	0.304	0.094	0.155	0.511
	$\rho_{(e:old,\ t6)}$	0.474	0.102	0.287	0.668
	$\rho_{(e:old,\ t7)}$	0.292	0.084	0.157	0.478
	$\rho_{(e:old,\ t8)}$	0.512	0.100	0.323	0.697
	$\rho_{(e:old,\ t9)}$	0.672	0.109	0.437	0.844
Maternal size/age ω = 0.33	$\varphi_{(e:\ young)}$	0.855	0.048	0.735	0.926
	$\varphi_{(e:\ old)}$	0.957	0.019	0.900	0.983
	$\rho_{(t1)}$	0.098	0.094	0.014	0.463
	$\rho_{(t2)}$	0.186	0.076	0.079	0.378
	$\rho_{(t3)}$	0.160	0.066	0.068	0.334
	$\rho_{(t4)}$	0.000	0.000	0.000	0.000
	$\rho_{(t5)}$	0.343	0.095	0.185	0.545
	$\rho_{(t6)}$	0.509	0.100	0.322	0.694
	$\rho_{(t7)}$	0.319	0.085	0.178	0.503
	$\rho_{(t8)}$	0.537	0.098	0.349	0.716
	$\rho_{(t9)}$	0.689	0.105	0.460	0.852

	φ(pe: 2017/2018)	0.937	0.034	0.829	0.979
	φ(pe: 2018/2019)	0.806	0.057	0.670	0.895
	ρ(pe: 2017/2018, t1)	0.270	0.233	0.035	0.790
	ρ(pe: 2017/2018, t2)	0.288	0.175	0.070	0.684
	ρ(pe: 2017/2018, t3)	0.212	0.135	0.053	0.566
	ρ(pe: 2017/2018, t4)	0.000	0.000	0.000	0.000
	ρ(pe: 2017/2018, t5)	0.196	0.126	0.049	0.538
	ρ(pe: 2017/2018, t6)	0.557	0.138	0.297	0.790
	ρ(pe: 2017/2018, t7)	0.278	0.109	0.118	0.527
	ρ(pe: 2017/2018, t8)	0.531	0.129	0.291	0.758
Interannual variability	ρ(pe: 2017/2018, t9)	0.563	0.137	0.302	0.794
	ρ(pe: 2018/2019, t1)	0.000	0.000	0.000	0.000
	ρ(pe: 2018/2019, t2)	1.000	0.000	1.000	1.000
	ρ(pe: 2018/2019, t3)	0.232	0.146	0.057	0.600
	ρ(pe: 2018/2019, t4)	0.595	0.218	0.200	0.896
	ρ(pe: 2018/2019, t5)	0.000	0.000	0.000	0.000
	ρ(pe: 2018/2019, t6)	0.177	0.165	0.023	0.664
	ρ(pe: 2018/2019, t7)	0.000	0.000	0.000	0.000
	ρ(pe: 2018/2019, t8)	1.000	0.000	0.000	1.000
	ρ(pe: 2018/2019, t9)	0.310	0.269	0.037	0.841

Model notation: ‘t’ = time interval (fortnightly as follows: from t1 which corresponded to the second fortnight of September to t9 which corresponded to the first fortnight of February); ‘e’ = female age (hatchlings from young and old females), ‘pe’ = period (hatchlings from 2017/2018 and 2018/2019).

Figure S1. Diagram of the monitoring effort exerted tracking hatchlings to estimate the survival rates (in blue sampling days). The study period encompasses six months, the end of September to February, during the two periods: a) 2017/2018 and b) 2018/2019. The effort was higher at the beginning of the study to mark all the new individuals when they were born and highly active and detectable. The data for the model analysis was collected fortnightly, and tracking individuals that were coincident in the same weeks were grouped to increase the sample size.

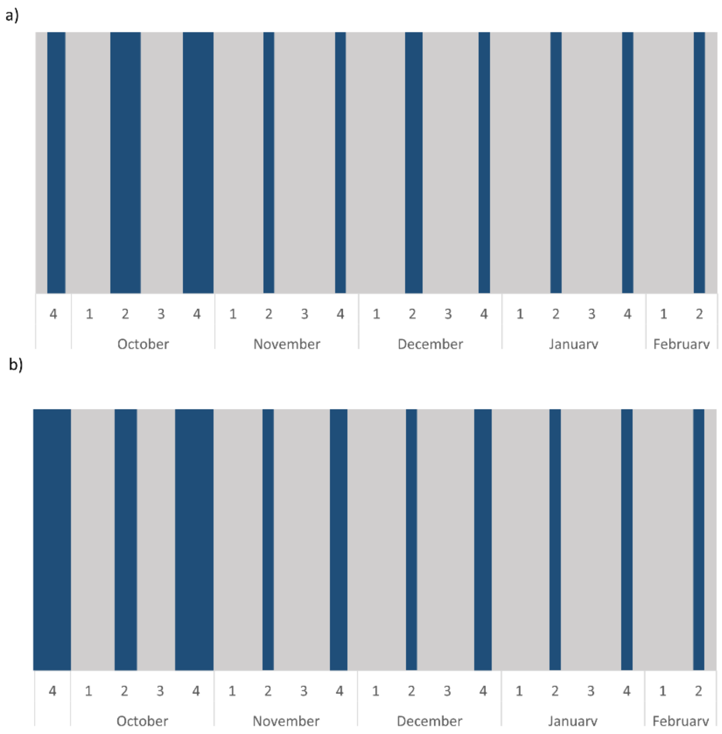

Supplement: Supplementary file 1 [file animals-11-00467-s001.pdf]
